# A Specific Male Olfactory Sensillum Detects Behaviorally Antagonistic Hairpencil Odorants

**DOI:** 10.1673/031.007.0401

**Published:** 2007-01-22

**Authors:** N. K. Hillier, D. Kelly, N. J. Vickers

**Affiliations:** Department of Biology, University of Utah, Room 201 South Biology, Salt Lake City, Utah, USA, 84112

**Keywords:** *Heliothis virescens*, Lepidoptera, courtship, behavioral antagonist, cobalt-lysine staining, antennal lobe, olfactory receptor neuron

## Abstract

Within insect species, olfactory signals play a vital role in communication, particularly in the context of mating. During courtship, males of many moth species release pheromones that function as aphrodisiacs for conspecific females, or repellants to competing conspecific males. The physiology and antennal lobe projections are described of olfactory receptor neurons within an antennal sensillum present on male *Heliothis virescens* F. (Lepidoptera: Noctuidae) moths sensitive to conspecific male *H. virescens*-produced pheromone components. Olfactory receptor neurons responded to hexadecanyl acetate and octadecanyl acetate hairpencil components, and Z11-hexadecenyl acetate, an odorant used by closely related heliothine species in their female produced pheromone, which is antagonistic to male *H. virescens* responses. This acetate-sensitive sensillum appears homologous to a sensillum type previously described in females of this species, sharing similar physiology and glomerular projection targets within the antennal lobe. Wind tunnel observations indicate that *H. virescens* hairpencil odors (hexadecanyl acetate, octadecanyl acetate) function to antagonize responses of conspecific males following a female sex pheromone plume. Thus, male-male flight antagonism in *H. virescens* appears to be mediated by this particular sensillum type.

## Introduction

Chemical signals represent an important source of information from an organism's environment. In many species of moth, conspecific pheromone signals produced by females have been shown to elicit upwind flight and mating attempts by males (Cardé and Baker 1984; Kennedy and Marsh 1974; Kennedy et al. 1980; Kennedy et al. 1981; Roelofs et al. 1974). Additionally, behavioral studies have shown that males and females of many species often engage in courtship behaviors once the male arrives in the proximity of the female (Heath et al. 1992; Jacquin et al. 1991; Raina and Stadelbacher 1990; Teal et al. 1981). Chemical analyses have demonstrated that males of several moth species release odors during courtship that have aphrodisiac effects on female conspecifics and repellent effects on the female-search behavior of conspecific males (Birch 1975; Birch and Hefetz 1987; Birch et al. 1990; Hillier and Vickers 2004). The sensory physiology of male antennal olfactory receptor neurons tuned to female-produced sex pheromone components has been well studied, but no reports have documented chemosensory cells tuned to distinctively male-produced odors in male moths (Hildebrand and Shepherd 1997; Christensen et al. 1991).

**Table 1.**
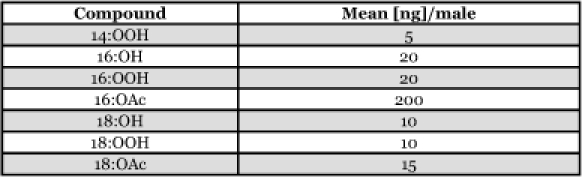
Male *H. virescens* hairpencil composition (adapted from Hillier and Vickers 2004)

In the tobacco budworm, *Heliothis virescens* F. (Lepidoptera: Noctuidae), males release a blend of 16 and 18 carbon chain acetates, alcohols and carboxylic acids from eversible abdominal scent brushes (hairpencils) during courtship with conspecific females (Table 1; Hendricks and Shaver 1975; Raina and Stadelbacher 1990; Teal and Tumlinson 1989). The specific ratio of components within this blend is required for mate acceptance by females, allowing for discrimination between appropriate suitors and males of other species with similar hairpencil effluvia (Hillier and Vickers 2004). Previous work has demonstrated that adult male *H. virescens* moths engage in fewer mating attempts with females when conspecific hairpencil extract is presented simultaneously on a filter paper disc. This behavioral observation suggested that release of hairpencil odors by *H. virescens* males serves a dual role during courtship: producing quiescence in courted females to facilitate mating, and repulsion of competing males (Hillier and Vickers 2004).

Within the insect antennal lobe, olfactory receptor neurons with similar odorant specificity synapse with antennal lobe neurons in distinct bundles of neuropil known as glomeruli (Berg et al. 1998; Hansson et al. 1992). In *H. virescens* males, the macroglomerular complex is a cluster of 4 sexually-dimorphic glomeruli present at the base of the antennal nerve specialized for processing female-produced pheromone components, as well as pheromones released by closely related species (Berg et al. 1998; Christensen et al. 1995; Vickers et al. 1998). Female *H. virescens* do not have a macroglomerular complex but instead have two enlarged ‘female-specific’ glomeruli at the base of the antennal nerve (the central and medial large female glomeruli; Berg et al. 2002; Hillier et al. 2006). Recently, the odorant affinity of olfactory receptor neurons terminating in several sexually isomorphic glomeruli within the female *H. Virescens* antennal lobe was investigated through cut-tip sensillar recording with cobalt-lysine staining. Olfactory receptor neurons sensitive to hairpencil components, female sex pheromones and plant volatiles were characterized and their associations with specific glomeruli determined (Hillier et al. 2006).

The goal of the current study was to further investigate the physiological and behavioral effects of *H. virescens* hairpencil odorants on males of this species. Olfactory receptor neurons were analyzed for their responses to components of the male hairpencil blend using single sensillum recording, and projections to the antennal lobe were determined using a cobalt-lysine staining technique (Hansson et al. 1992; Kaissling 1974; Todd et al. 1995). Wind tunnel experiments were subsequently conducted to determine if physiologically active odorants had any effect upon pheromone-mediated upwind flight.

## Methods

### Insects

Male moths used for single sensillum recording and wind tunnel assays were obtained from the *H. Virescens* colony maintained at the University of Utah. Larvae were reared on a pinto bean diet (Shorey and Hale 1965) until pupation. Pupae were sorted by sex and separated into different environmentally controlled chambers (Percival Scientific, www.percival-scientific.com). Adults were kept at 25°C, 60% relative humidity, on a reversed light cycle (14L:10D) until eclosion.

### Chemicals

Odorants were selected for electrophysiology and behavioral experiments based on the previously identified hairpencil composition of male *H. virescens* (Table 1; Teal and Tumlinson 1989). Compounds found in the hairpencil glands were obtained from Dr. James Tumlinson (Department of Entomology, The Pennsylvania State University, University Park, PA), and Sigma Aldrich (www.sigmaaldrich.com).

Six odorants found in male hairpencil glands were tested for electrophysiological activity:

hexadecanyl acetate (16:OAc)
hexadecan-1-ol (16:OH)
hexadecanoic acid (16:OOH)
octadecanyl acetate (18: OAc)
octadecan-i-ol (18:OH)
octadecanoic acid (18:OOH)

Four components of female heliothine sex pheromones were also used in this study:

(Z)-11-hexadecenyl acetate (Z11-16:OAc), a behavioral antagonist to *H. virescens* males produced by *Heliothis subflexa* Guenée females (Vickers and Baker 1997). A type 1 sensillum on female *H. virescens* antennae was also found to respond to Z11-16:OAc, 16:OAc and 18:OAc (Hillier et al. 2006)
(Z)-11-hexadecenal (Z11-16:Ald), which is a major component of the female *H. virescens* sex pheromone blend;
(Z)-9-tetradecenal (Z9-14:Ald), which is a minor component of the female *H. virescens* sex pheromone blend, and is required in a mixture with Zn-16:Ald (100:5) to attract males;
(Z)-11-hexadecen-1-ol (Z11-16:OH), a behaviorally innocuous odorant found in female *H. virescens* pheromone gland extracts (Klun et al. 1979; Klun et al. 1980; Pope et al. 1982; Roelofs et al. 1974; Teal et al. 1986; Tumlinson et al. 1975; Tingle et al. 1978; Vetter and Baker 1983).

Female sex pheromone components were obtained from Bedoukian Research Inc. (www.bedoukian.com). Solutions were diluted as a decade series (ing-img) in hexane and stored at -20°C, with the exception of 16:OH, 18:OH 16:OOH and 18:OOH that solidify at room temperature at concentrations above 100 µg. Samples of stock solutions were confirmed as >95% purity by injection onto a Shimadzu GC 17A gas chromatograph (www.shimadzu.com) equipped with a 30 m × 0.25 mm ID DB-5 capillary column.

### Single sensillum recording

Electrophysiological characterization of sensilla was conducted using a cut-sensillum technique (Kaissling 1974). Male moths were secured with dental wax in 1ml disposable pipettes and mounted to a depression slide. The tip of the antenna was fastened horizontally to the side of the slide using water-soluble correction fluid (Liquid Paper^®^, Paper Mate). Once mounted, a reference silver-chloride electrode was inserted into the contra-lateral eye. Moths were placed under a compound microscope and antennal sensilla observed at 20 X. Sexually isomorphic short trichoid sensilla (length 30–50 µm and diameter 2 µm) were selected from the mid-ventral side of an antennal segment and cut using a resonating glass capillary mounted to a peizo crystal attached to a function generator (Gödde 1989; Hillier et al. 2006). Once cut, a saline-filled glass capillary electrode was positioned over the sensillum using a micromanipulator and electrical contact made with the sensillum.

Sensilla were screened using the odorant array for physiological activity. Following identification of physiologically active sensilla, if signal-to-noise was still satisfactory, dosage-response series were performed with stimuli ranging from 1 µg to 1 mg. Signals were amplified (1000X, ER-1®, Cygnus Technology, www.cygnustech.com), filtered (HUMBUG^®^, Quest Scientific, www.quest-sci.com), and data recorded directly to a computer using Labview 6.0^©^ software.

### Odorant stimulation

Stimulus cartridges were made by applying 10µl of an odorant to a 5 × 30 mm piece of filter paper.

After the hexane had evaporated, the filter paper was inserted into a 1 ml syringe. Stimulus concentrations in each cartridge ranged between 100 ng and 1 mg. A continuous flow (1 L/min) of charcoal-filtered, humidified air was blown over the antenna. Stimulus pulses were created by switching a continuous flow between an exhaust port and an odor cartridge using a solenoid valve and valve driver (Parker-Hannafin, www.parker.com). Continuous and stimulus air converged in a mixing chamber (50 mm long × 5 mm inner diameter), the exit of which was positioned 10 mm from the insect's antenna. Stimulation was automatically controlled using custom software written in Labview© 6.0 (National Instruments, www.ni.com/labview). For each sensillum tested, odorants were presented in random order, with 60 seconds elapsed between each stimulation to prevent adaptation. Sensilla were screened for sensitivity using 100 µg of each stimulus, and hexane was used as a control stimulus. Stimulation was a series of 3 × 200 ms puffs separated by 1 second each. 2 seconds pre-stimulation and 1 second post-stimulation were recorded, resulting in 6 seconds total recording time. A main-effects ANOVA was used to determine significant differences in spike frequency with concentration nested within odorant. Means were separated using Fisher's LSD Test (p<0.05).

### Staining of sensory projections and 3-D reconstructions

In many cases, once the physiological activity of a sensillum was characterized, attempts were made to identify the axonal projections of olfactory receptor neurons within the sensillum to glomeruli in the antennal lobe. A glass capillary filled with a solution of cobalt-lysine (2.38 g cobaltous chloride with 5 g L-lysine in 20 ml of distilled water, lowered to a pH of 7.2–7.4 using concentrated HCl) was placed over the cut-tip of the sensillum. The sensillum was stimulated with a 100 ms, 0.5 Hz ‘puff’ of an excitatory odorant for 10 minutes and the glass capillary was left in contact with the sensillum for a total of one hour.

Insects were placed in a Petri dish with a piece of moistened paper towel, and put in a 4°C refrigerator. After 48 hours, the brains were removed and fixed in a 100% Ethanol: Acetic acid: 38% Formaldehyde (8:1:2) solution for 24 hours, and subsequently subjected to silver intensification (Timm 1958) for 20–30 minutes. Brains were serially dehydrated, embedded in Spurrs resin (Electron Microscopy Sciences, Ft. Washington, PA), sectioned at µm and mounted on microscope slides. Before coverslipping, sections were counterstained using a modified solution of Lee's Methylene Blue-Basic Fuchsin solution (methylene blue +Azure II in borate): 0.5% Basic Fuchsin in 95% ethanol: 100% ethanol = 1:2:1 (Lee et al. 2006). Sections were examined at 20–40 X and digital images were taken using an attached Optronics Microfire© (www.optronics.com) camera.

Digital images were taken at 20X as serial sections through the antennal lobes to identify the glomerular targets of cobalt-lysine stained olfactory receptor neurons from selected sensilla. TIFF files were imported to AMIRA 2.3™ (Indeed GmbH, Berlin; http://www.amiravis.com) and subjected to image segmentation to delineate boundaries of the antennal lobe and individual glomeruli, along with the stained olfactory receptor neurons. This permitted individual labeling and 3D reconstruction of the antennal lobe structure. Reconstructions were compared to the *H. virescens* antennal lobe atlas (Berg et al. 2002). Selected images were exported to Adobe Photoshop to adjust contrast and brightness.

### Wind tunnel experiments

Cohorts (3–7 days old) of male moths were selected for wind tunnel experimentation. The characteristics of the wind tunnel have been described previously (Vickers 2002). Briefly, the working section of the wind tunnel was 2.5 × 1.14 × 1.14 m (L × H x W). Illumination was provided by one red and one white incandescent bulb, each controlled by a rheostat. Environmental parameters in the wind tunnel were as follows: wind speed, 0.47 – 0.60 m/s; temperature, 21.1-22.0°C; relative humidity, 25.4%–45.5%.

Individual male insects were placed in wire cages (3 cm diameter × 5 cm high), within a plastic container before scotophase on the day of experimentation. Containers were returned to an environmental chamber until the 2^nd^ hour of scotophase. Insects were then moved to the wind tunnel room, and individual cages were placed (inverted to prevent escape) at the side of the wind tunnel to allow a period of acclimation to the conditions therein. During behavioral testing, a filter paper disc loaded with an odor blend was attached to an alligator clip on a metal rod and introduced to the wind tunnel such that the odor source was 1.5 m upwind from the moth ‘take-off’ platform and 24 cm above the wind tunnel floor. The take-off platform was located 50 cm upwind from the exhaust vent, centrally placed to intersect the pheromone plume.

**Figure 1.**
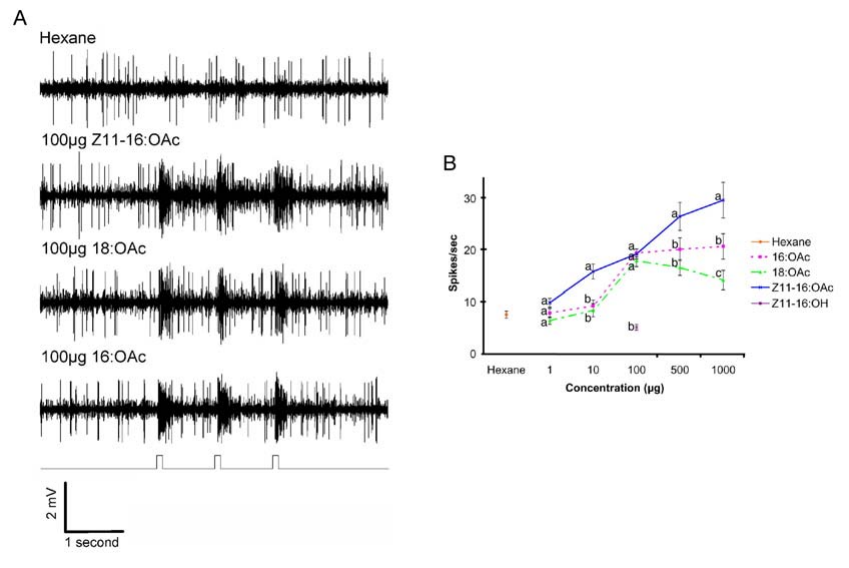
A: Single sensillum responses (original spike trains) from a male *H. virescens* antenna to stimulation with a hexane blank, Z11-16:OAc, 18:OAc and 16:OAc. B: Mean (± SE) dosage response curves from acetate-sensitive sensilla (1-1000 µg; n = 28 sensilla from 28 moths). No response noted to screening with hexane or Z11-16:OH. A total of 275 sensilla from 41 moths were screened for odor-evoked activity in response to hairpencil components. Data points at the same concentration represented by different letters are significantly different (F_12, 576_=19.45; Fisher's LSD, p<0.05).

Wind tunnel assays were used to determine any effect of physiologically active hairpencil odorants on male flight responses to female sex pheromones. Specifically, 16:OAc and 18:OAc were tested but not Zn-16:OAc as its behavioral role (inhibitory) has been described previously (Vickers and Baker 1996). Control trials were conducted using a mixture of µg Z11-16:Ald + 50 ng Z9-14:Ald [‘*H. virescens* 2-mix’, a known attractant for male *H. virescens*] admixed on 1-cm diameter filter paper discs (No. 4, Whatman; (Pope et al. 1982; Vetter and Baker 1983). To test the effects of hairpencil odorants, 16:OAc and 18:OAc were admixed with the ‘*H. virescens* 2-mix’ on filter paper discs at increasing dosages: 10 ng, 100 ng and µg (or 1, 10 and 100% relative to the concentration of Z11-16:Ald in the mixture). Hexane was evaporated from filter paper in the fume hood before testing. Experiments were conducted with groups of 10–20 insects per day. A single odor-concentration treatment was tested each day, along with 5–10 insects flown to the control pheromone mixture.

For each trial, moths were released by inverting their cage and placing it on the ‘take-off’ platform to permit the moth to exit. Insect activity was quantified using the following behavioral sequence:

Take Flight: Moth ‘activates’, begins wing fanning and engages in flight.
Upwind Flight: Moth locates plume, and begins characteristic counterturning flight towards odor source.
75cm: Moth continues upwind counterturning flight beyond half the distance between the take-off platform and the odor source.
5cm: Moth continues upwind counterturning flight to within 5cm of the odor source.
Source Contact: Moth lands on the odor source.

**Figure 2.**
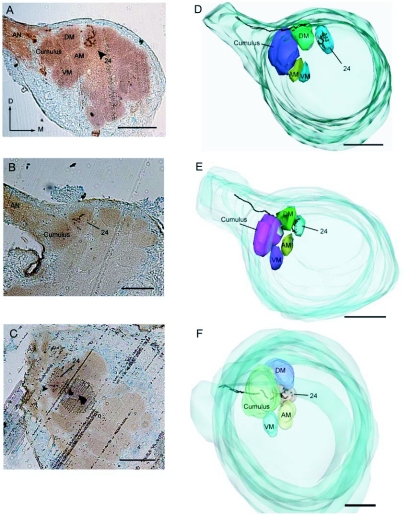
Male *H. virescens* olfactory receptor neurons stained from sensilla sensitive to male-produced sex pheromone components (16:OAc, 18:OAc), showing the antennal lobe (AL) target (glomerulus 24) relative to the 4 macroglomerular complex glomeruli: cumulus, dorsomedial glomerulus (DM), ventromedial glomerulus (VM), and anteromedial glomerulus (AM). A-C: Cobalt-lysine stains from acetate-sensitive sensilla revealed uniglomerular arborizations in glomerulus 24, medial to the MGC (each stain from a different moth preparation). D-F: Digital reconstructions from A-C showing the stained neuron and glomerular target in relation to the MGC. Orientation is similar between all micrographs and reconstructions. Arrowheads indicate location of the stain in the micrographs. Dorsal *D;* Medial *M;* antennal nerve *AN.* Scale bars = 100 µm.

**Figure 3.**
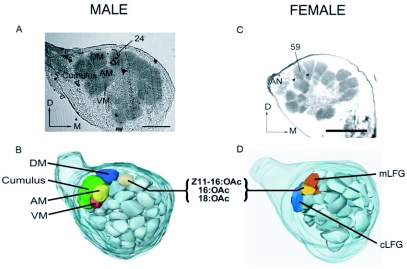
Comparison between male and female *H. virescens* glomerular projections of acetate-sensitive olfactory receptor neurons to the antennal lobe: A: Cobalt lysine stain from an acetate-sensitive sensillum projecting to glomerulus 24 in male *H. virescens.* B: Digital reconstruction of the male antennal lobe showing the position of glomerulus 24 relative to the macroglomerular complexes (cumulus, dorsomedial glomerulus (DM), ventromedial glomerulus (VM), and anteromedial glomerulus (AM)) and other isomorphic glomeruli in the antennal lobe. C: Cobalt lysine stain from an acetate-sensitive sensillum (Type 1) projecting to glomerulus 59 in female *H. virescens.* D: Digital reconstruction of the female antennal lobe showing the position of glomerulus 59 relative to other glomeruli. Female-specific Large Female Glomeruli (LFGs; central large female glomerulus: cLFG, and medial large female glomerulus: mLFG) have been labeled in reconstruction to show glomerulus 59 which lies anterior to the LFGs). Female data in C and D from Hillier and Vickers (2004). Arrowheads indicate location of the stain in the micrographs. Dorsal, *D;* medial, *M;* Scale Bars = 150µm.

Percentage of males engaging in each behavioral step were compared using a χ^2^ 2 × 2 test of independence with Yates' correction. Results were considered statistically significant if p <0.05 (Sokal and Rohlf 1995).

## Results

Screening of various hairpencil components through single sensillum recording revealed a sensillum ‘type’ containing an olfactory receptor neuron that responded selectively to 16: OAc, 18:OAc and Z11-16:OAc (n = 28; Figure 1). Uniform spike amplitudes from recordings suggested that this sensillum housed a single olfactory receptor neuron. A single recording was made from a sensillum type containing an olfactory receptor neuron that responded exclusively to 16:OH (n = 1).

The olfactory receptor neuron present in the acetate-sensitive sensillum responded in a phasic manner to repeated stimulation (Figure 1A). Dosage-response curves indicated that the olfactory receptor neuron was most sensitive to Z11-16:OAc, followed by 16:OAc and 18:OAc, at 10 µg, 500 µg and 1000 µg stimulus concentrations (F_12, 576_=19.45, p<0.05; Figure 1B). Dosage-response was similar for 16: OAc and 18: OAc at all concentrations tested, excepting 1000 µg (Figure 1). No response was noted to control stimulation with hexane alone. Furthermore, these sensilla lacked a response to Z11-16:OH, and were therefore distinct from ‘Type C’ sensilla previously described from this species (*H. virescens* Type C sensilla contain two olfactory receptor neurons: one sensitive to Z11-16:OAc and a second sensitive primarily to Z11-16:OH and weakly to Z9-14:Ald; Almaas and Mustaparta 1991; Baker et al. 2004; Berg et al. 1998). Moreover, Type C sensilla olfactory receptor neurons are not sensitive to either 16:OAc or 18:OAc (unpublished observations).

**Figure 4.**
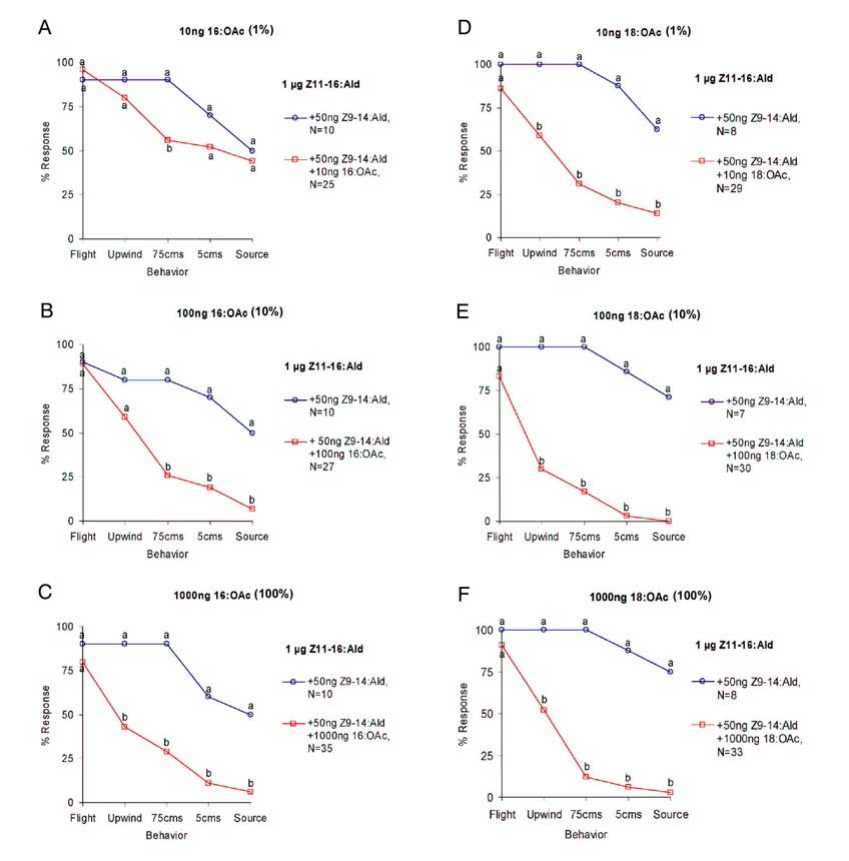
Behavioral flight responses of male *H. virescens* to an attractive pheromone mixture [1µg Z11-16:Ald + 50ng Z9-14:Ald] alone and combined with varying dosages of either 16:Oac: (A) 10 ng, (B) 100 ng and (C) 1000 ng; or 18:OAc: (D) 10 ng, (E) 100 ng and (F) 1000 ng. Data points represented by different letters are significantly different (χ^2^ 2 × 2; p<0.05).

Cobalt lysine staining of olfactory receptor neurons within this type of sensillum revealed consistent axonal projections to a glomerulus near the base of the antennal nerve (N=13 stains/23 attempts; Figure 2). This glomerulus was situated medial and dorsal to the macroglomerular complex, in a position corresponding to glomerulus 24, described in the male *H. virescens* antennal lobe atlas (Berg et al. 2002). The physiological response and glomerular projection of this hairpencil-sensitive olfactory receptor neuron corresponded with a sensillar type present in female *H. virescens* (type 1) which responded selectively to 16:OAc, 18:OAc and Z11-16:OAc and also projected to a glomerulus (#59 according to the female antennal lobe atlas; Berg et al. 2002), near the base of the antennal nerve (Hillier et al. 2006; Figure 3).

Wind tunnel experiments indicated that addition of 16:OAc or 18:OAc to a synthetic female pheromone attractant mixture (100:5 of Z11-16:Ald and Z9-14:Ald) produced a significant decrease in upwind flight and contact with a filter paper odor source (Fig. 4A-B). Male moths showed an overall reduction in activation, and prematurely ceased upwind flight when following the odor plume. This effect was concentration-dependent for both hairpencil components. Ratios of 18:OAc and 16:OAc as low as 1% and 10% respectively, of the primary odorant in the mixture (1µg Z11-16:Ald) were effective in suppressing flight behavior and source location.

## Discussion

These Results provide the first description of an olfactory sensillum present on a male insect sensitive to intra-sexual odor cues. *H. virescens* male hairpencil odors may function as an anti-aphrodisiac during courtship, repelling competing conspecific males, as upwind flight to synthetic female sex pheromone is inhibited by coincident presentation of 16:OAc and 18:OAc. The presence of uniglomerular projections and uniform spike amplitudes also suggests that this sensillum houses a single 18:OAc/16:OAc/Z11-16:OAc sensitive olfactory receptor neuron that principally regulates this behavioral effect. It is possible that additional rare sensillar types housing additional olfactory receptor neurons (such as the single 16:OH sensitive sensillum) are also present on the antenna (sensitive to the hairpencil odorants tested) but no others were noted in this study.

When comparing the sensitivity of this olfactory receptor neuron to each of the tested odorants (Figure 1B), it is important to consider their relative volatilities. Filter paper cartridges were loaded at similar concentrations, however the relative molecular weights and vapor pressures will affect the emission rates and volume of odorant delivered in each stimulus ‘puff’ (Cossé et al. 1998). 18:OAc has a considerably lower vapor pressure (6.1×10^-5^ mm Hg) than either 16:OAc (2.7×10^-4^ mm Hg) or Z11-16:OAc (3.9×10^-4^ mm Hg), and therefore was delivered at a much lower concentration relative to it loading in the stimulus cartridge (Koutek et al. 1992; PhysProp Online™ 2006). Therefore, this olfactory receptor neuron is most likely primarily sensitive to 18: OAc, and secondarily to 16:OAc/Z11-16:OAc, based on dosage response curves (Figure 1). This is further supported by the wind tunnel assay wherein antagonism of upwind flight was more pronounced in conjunction with 18:OAc presentation (Figure 4). It remains unclear, however, whether 18:OAc or 16:OAc is more important in mediating male-male interactions, as the concentration of 16: OAc is much higher in male *H. virescens* hairpencil gland extracts (the natural concentration of each in airborne effluvia is unknown; Teal and Tumlinson, 1989; Table 1).

The proximity of this glomerulus to the macroglomerular complex may enable integration and cross-glomerular interactions between glomeruli during processing of attractive odorants, such as Z11-16:Ald or Z9-14:Ald, and antagonistic cues such as those in the hairpencil blend. Previous studies have found that Z11-16:OAc, an antagonistic odorant released in the pheromone blends of closely related species, is processed in an anteromedial glomerulus of the macroglomerular complex (Figure 3; Vickers et al. 1998). Evidence from the current study now indicates that at least two glomeruli in relatively close proximity are activated by the presence of Z11-16:OAc. This glomerular organization may represent an important facet in the general combinatorial coding of Z11-16:OAc through activation of both glomeruli simultaneously. Alternatively, Z11-16:OAc may be important to male *H. virescens* under different behavioral contexts, and hence is represented within two distinct locations in the antennal lobe.

This study also presents the possibility that in this species a functional division of the antennal lobe exists, wherein glomeruli near the base of the antennal nerve serve to process odors produced by both males and females. The functional role of the sexually dimorphic macroglomerular complex structure in all moth species appears to be for processing female sex pheromones and odorants present in the blends of other sympatric species (Christensen and Hildebrand 2002; Hansson and Christensen 1999). Our current Results suggest that a subgroup of glomeruli at the base of the antennal nerve, including the macroglomerular complex, may be responsible for processing both male and female produced pheromones. Whereas the macroglomerular complex is likely a derived structure, regional specialization of isomorphic glomeruli near the base of the antennal nerve may be the ancestral state for macroglomerular complex development (Hansson and Christensen 1999). Furthermore, this provides an indication of specialization of glomerular clusters throughout the antennal lobe, wherein glomeruli receiving input from odors of a similar behavioral meaning (e.g. male and female pheromones, host plant odors), might be localized in close proximity within the antennal lobe.

Physiological and anatomical similarities were found between males and females in the odorant response and axonal projection patterns of olfactory receptor neurons within this sensillum type. We have previously identified a sensillar type on female *H. virescens* which contained an olfactory receptor neuron responsive to 18:OAc, 16:OAc and Z11-16:OAc (type 1) and projected to a glomerulus near the base of the antennal nerve (Figure 3; Hillier et al. 2006). This suggests a degree of homology in peripheral and central processing of these hairpencil odors between the sexes. This result concurs with the overall similarity in antennal lobe structure observed between sexes in this species, and with results from imaging studies that have documented similar odor-evoked activation patterns in the antennal lobe of male and female moths (Berg et al. 2002; Skiri et al. 2004; Galizia et al. 2000). Similar glomerular topology between males and females may provide an anatomical ‘baseline’ for the development of sexually dimorphic antennal lobe structures, such as the macroglomerular complex.

In many species of Lepidoptera, such as members of the Arctiidae and Danaidae, hairpencil composition is related directly to host plant consumption and sequestration of compounds during larval and adult stages (Birch and Hefetz 1987). Because of the importance of oviposition upon appropriate host plants in many of these species, females are predicted to have preexisting olfactory receptor neuron sensitivity, and a behavioral attraction to these volatiles (Bernays and Chapman 1994; Hillier et al. 2006). In *H. virescens,* however, male hairpencil composition is more similar to female *H. virescens* pheromone chemistry than the known chemistry of host plants (Teal and Tumlinson 1989; Klun et al. 1979; Klun et al. 1980; Pope et al. 1982; Roelofs et al. 1974; Teal et al. 1986; Tumlinson et al. 1975; Tingle et al. 1978; Vetter and Baker 1983). Therefore the relative proximity of glomeruli processing male and female sex pheromones provides evidence that the evolution of the antennal lobe and macroglomerular complex organization may be tied to the emergence of olfactory-mediated courtship behavior in *H. Virescens.*

It is noteworthy that the behavioral outcome of detecting these odorants might be relatively similar between the sexes — an overall suppression in activity. In female *H. virescens,* exposure to hairpencil pheromone results in quiescence that facilitates mating, in males, it inhibits upwind flight toward a calling female (Hillier and Vickers 2004). Further work will be required, however, to clarify how similar hairpencil odor processing is between the sexes, despite the behavioral outcome.

For the wind tunnel assays, aliquots of 16:OAc and 18: OAc were applied, along with the agonistic pheromone blend, directly to the filter paper odor source resulting in co-emission of pheromone blend and test odorant. This, however, may not precisely represent the natural circumstance, wherein a courting male would expose hairpencils adjacent (2–10 mm) to the calling female. Previous work on *Helicoverpa zea* has indicated that male moths exhibited weaker antagonism when an antagonist was presented on an adjacent odor source (1 mm upwind or downwind), compared with application of agonistic and antagonistic odorants on the same odor substrate (Baker et al. 1998; Fadamiro and Baker 1997). Male-male behavioral responses to 16: OAc and 18: OAc therefore might also be affected by such fine scale integration of agonistic and antagonistic odorant plumes. Consequently, male *H. virescens* hairpencil components released during normal courtship may result in differing behavioral outcomes with coincident odor delivery, than those observed herein.

This discovery provides an insight into olfactory processing of a male sex pheromone by conspecific males, as well as a potential new prospect for management of *H. virescens* pest populations.

## Abbreviations

Z11-16: Ald (Z)-11-hexadecenal
Z9-14: Ald (Z)-9-tetradecenal
Z11-16: OAc (Z)-11-hexadecenyl acetate
Z11-16: OH (Z)-11-hexadecen-1-ol
16: OH hexadecan-1-ol
18: OH octadecan-1-ol
16: OAc hexadecanyl acetate
18: OAc octadecanyl acetate
16: OOH hexadecanoic acid
18: OOH octadecanoic acid
